# A simple method to combine multiple molecular biomarkers for dichotomous diagnostic classification

**DOI:** 10.1186/1471-2105-7-442

**Published:** 2006-10-10

**Authors:** Manju R Mamtani, Tushar P Thakre, Mrunal Y Kalkonde, Manik A Amin, Yogeshwar V Kalkonde, Amit P Amin, Hemant Kulkarni

**Affiliations:** 1 Lata Medical Research Foundation, Nagpur, India; 2 University of North Texas Health Science Center, Fort Worth, Texas, USA

## Abstract

**Background:**

In spite of the recognized diagnostic potential of biomarkers, the quest for squelching noise and wringing in information from a given set of biomarkers continues. Here, we suggest a statistical algorithm that – assuming each molecular biomarker to be a diagnostic test – enriches the diagnostic performance of an optimized set of independent biomarkers employing established statistical techniques. We validated the proposed algorithm using several simulation datasets in addition to four publicly available real datasets that compared i) subjects having cancer with those without; ii) subjects with two different cancers; iii) subjects with two different types of one cancer; and iv) subjects with same cancer resulting in differential time to metastasis.

**Results:**

Our algorithm comprises of three steps: estimating the area under the receiver operating characteristic curve for each biomarker, identifying a subset of biomarkers using linear regression and combining the chosen biomarkers using linear discriminant function analysis. Combining these established statistical methods that are available in most statistical packages, we observed that the diagnostic accuracy of our approach was 100%, 99.94%, 96.67% and 93.92% for the real datasets used in the study. These estimates were comparable to or better than the ones previously reported using alternative methods. In a synthetic dataset, we also observed that all the biomarkers chosen by our algorithm were indeed truly differentially expressed.

**Conclusion:**

The proposed algorithm can be used for accurate diagnosis in the setting of dichotomous classification of disease states.

## Background

In spite of a plethora of available choices [1-4] to statistically analyze the high-dimension data derived from gene microarrays or serum proteomic profiles, a single method of choice remains elusive. Two main objectives motivate such analyses: first, to identify novel biomarkers that characterize specific disease states so as to gain biological and therapeutic insights and second, to identify patterns of expression that will discriminate among disease states and aid in diagnostic classification. Clearly, the statistical methods suited to achieve one objective may differ from the methods appropriate for the other purpose – not only in terms of the procedural assumptions and details but also in terms of the results. Several extensive reviews deal with this issue [1,5-10].

For the purpose of diagnostic classification using biomarker data, a common statistical option is to use linear discriminant functions [11,12]. However, this choice is not straightforward because of an important asymmetric disposition of the typical datasets. For example, the number of biomarkers (usually several thousands) greatly exceeds the number of samples (usually in hundreds or less). Each biomarker, therefore, must be assessed for its potential association with the disease status leading to a problem of multiple comparisons [13]. Consequently, while on the one hand a few highly significant associations may mask other important associations; on the other hand noisy, false positive associations can be detected. A successful use of linear discriminant analyses therefore rests on a proper choice of a finite but informative subset of the biomarkers.

A natural approach to supervised diagnostic classification using such datasets would be to assume expression of each biomarker as a diagnostic test [14,15]. Since the expression levels of many of the biomarkers can be expected to be correlated with each other [15], the problem in diagnostic classification is that of finding an optimum combination of the correlated diagnostic tests that will maximize the discriminatory performance. The formal optimization methods available for this problem are however constrained to a limited number of diagnostic tests[16] An analysis of a dataset of n biomarkers will need to consider a total of 2^n^-1 combinations of the biomarkers, which can demand immense computation time in the context of the microarray experiments. We believe, however, that a simpler solution to this problem exists, which can be arrived at by using classical statistical techniques. Here, we suggest a statistical algorithm to circumvent these problems of data dimension and asymmetry and using four datasets of differing nature demonstrate the use of the proposed technique for the purpose of dichotomous diagnostic classification.

## Results

### Proposed statistical algorithm

**Figure 1:**
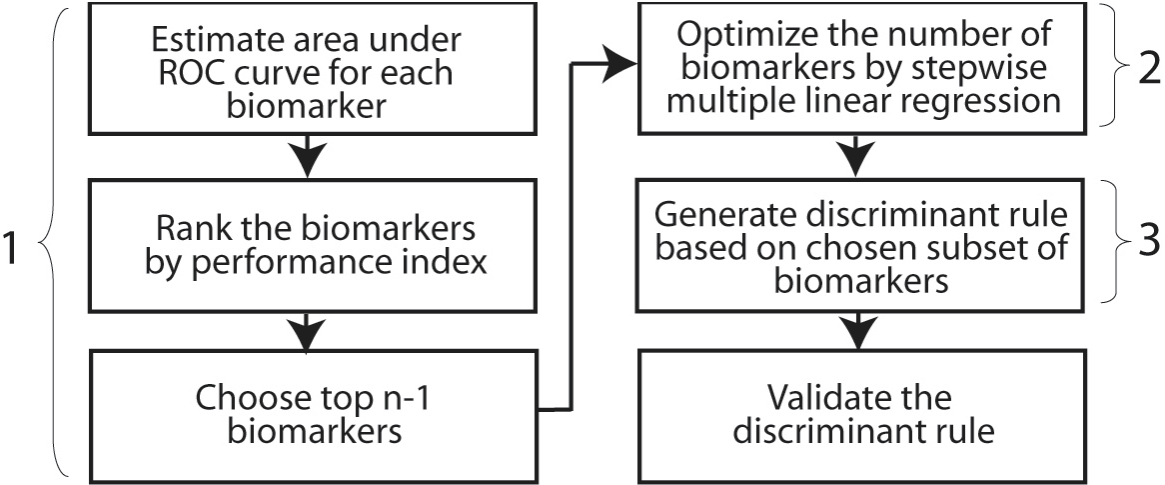
**The statistical algorithm used in the present study**. Numbers correspond to the steps described in the text. Steps 1–3 were used on a training subset. The training subset was randomly chosen for the OvCa dataset while for the other two datasets, the training sets used by primary authors were used. Validation was done separately in the training and test subset within each dataset.

Contingent upon the assumption that each biomarker can be considered as a diagnostic test, we propose a three-step procedure (Fig. 1) to arrive at an optimum combination of the biomarkers that will have a high degree of diagnostic accuracy. The proposed algorithm consists of three steps: screening the biomarkers individually based on the Performance Index (P_i_) which is a function of the estimated area under the receiver characteristic curve (AUC), using stepwise multiple regression analysis to select the top ranked n-1 biomarkers, and then combining the selected biomarkers using a linear discriminant function. A detailed description of the algorithm and its implementation in Stata 7.0 statistical package (Stata Corp, College Station, Texas) is provided in the Methods section and in Section 1 of the Additional File (see additional file 1). To assess the validity of our proposed algorithm, we used five datasets with 2 diagnostic classes each – four real datasets [17-20] (abbreviated as OvCa, LuMe, LLML and BrCa and described in Table 1) and a synthetically generated dataset (abbreviated as Syn1) comprising 50 samples of each diagnostic class and 1000 genes (described in the Methods section below).

**Table 1:** The publicly available datasets used in the current study

**Characteristic**	**Dataset #1**		**Dataset #2**		**Dataset #3**		**Dataset #4**	
Authors	Petricoin et al, 2002 [19]		Gordon et al, 2002 [18]		Golub et al, 1999 [17]		van't Veer et al 2002 [20]	

Dataset alias	OvCa		LuMe		LLML		BrCa	

Biomarker	Proteomic mass spectra		Gene expression		Gene expression		Gene expression	

# Biomarkers	15,154		12,533		7,129		24,481	

Diagnostic classes	Ovarian cancer	Normal	Lung adeno-carcinoma	Mesothelioma	Acute lymphocytic leukemia	Acute myeloid leukemia	Metastasis within 5 years	Metastasis after 5 years

N (Total)	162	91	150	31	46	26	34	44
N (Training)	83	49	16	16	27	11	17	26
N (Test set)	79	42	134	15	19	15	17	18

### Results from step 1 of the proposed algorithm

**Figure 2:**
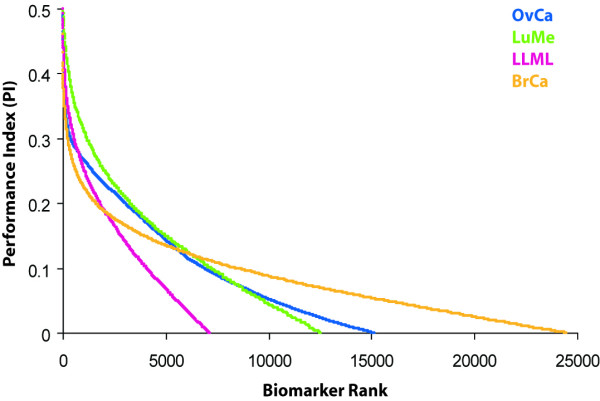
**The performance index (P_i_) derived from the area under the ROC curve of the biomarkers within each dataset**. The curves demonstrate that the diagnostic performance of the biomakers follows the Zipf's law. The colors for the four datasets are used consistently in Figure 3 and Supplementary Figures 1 and 2 (see additional file 1).

In the context of microarray experiments, the Zipf's law suggests that there will be only few genes with very highly differential expression levels [21-24]. Analogous to this interpretation of the Zipf's law, in our situation, we expected a small subset of biomarkers to be very highly discriminatory for diagnostic classification. Fig 2 shows that this indeed was the case. The proportion of biomarkers with performance index exceeding 0.4 (which corresponds to an AUC more than 0.9 or less than 0.1) was 0.31%, 2.36%, 1.25%, 0.03% and 11.1% in the OvCa, LuMe, LLML, BrCa and Syn1 datasets, respectively. This observation indicated that a combination of only few biomarkers is likely to be diagnostically sufficient.

Further, the OvCa dataset (as available from the source) was already normalized while the other three datasets used raw expression level values. As can be expected from the non-parametric nature of the area under the ROC curve statistic, this preprocessing (or the lack of it) of the data did not alter the diagnostic performance across the datasets. To explore this formally, we generated a synthetic dataset (Syn2) with 100 biomarkers and 100 subjects using raw as well as normalized expression values. The area under the ROC curve for the biomarkers – whether raw or normalized – was comparable (see Section 2 of additional file 1).

### Results from step 2 of the proposed algorithm

As there were 132, 32, 38 and 43 subjects (Table 1) in the training subsets of the OvCa, LuMe, LLML and BrCa datasets, respectively; we chose 131, 31, 37 and 42 biomarkers in the corresponding full models in these datasets. We observed that the number of biomarkers that were retained in the final model in stepwise multiple linear regression analyses was strikingly low. For example, in the OvCa dataset 18 (of 131), in the LuMe dataset 5 (of 31), in the LLML dataset 3 (of 37) and in the BrCa dataset 5 (of 42) biomarkers were retained in the final model. In spite of these small number of biomarkers, the regression model fit was excellent within each dataset (see Section 3 in additional file 1).

In the OvCa dataset, the mass-by-charge (M/Z) values identifying the 18 biomarkers retained in the final model were as follows: 2.8549, 25.4956, 25.6844, 25.7791, 28.7005, 42.4388, 220.7513, 243.4940, 245.2447, 434.6859, 463.1559, 463.5577, 463.9596, 464.3617, 619.0509, 8033.385, 8035.058, and 8038.405. In the LuMe dataset the following five probe sets were retained in the final model: junction plakoglobin, tumor-associated calcium signal transducer 2, adaptor-related protein complex 2, EGF-containing fibulin-like extracellular matrix protein 1 and polymerase I and transcript release factor. In the LLML dataset the three genes that were retained in the final model were LYN V-yes-1 Yamaguchi sarcoma viral related oncogene homolog, Calpain 2 and the Epb72 gene. In the BrCa dataset, the five genes that were retained in the final model were: NGFIA-binding protein-2 (NAB2), aurora kinase A interacting protein 1 (AURKAIP1), V-set domain containing T cell activation inhibitor 1 (VTCN1), zinc finger protein 473(ZNF473) and leucine-rich repeats and calponin homology (CH) domain containing 3 (LRCH3). The ROC curve for the diagnostic performance of each biomarker retained in the final model within each dataset is shown in Fig A1 (see additional file 1).

### Results from step 3 of the proposed algorithm

**Figure 3:**
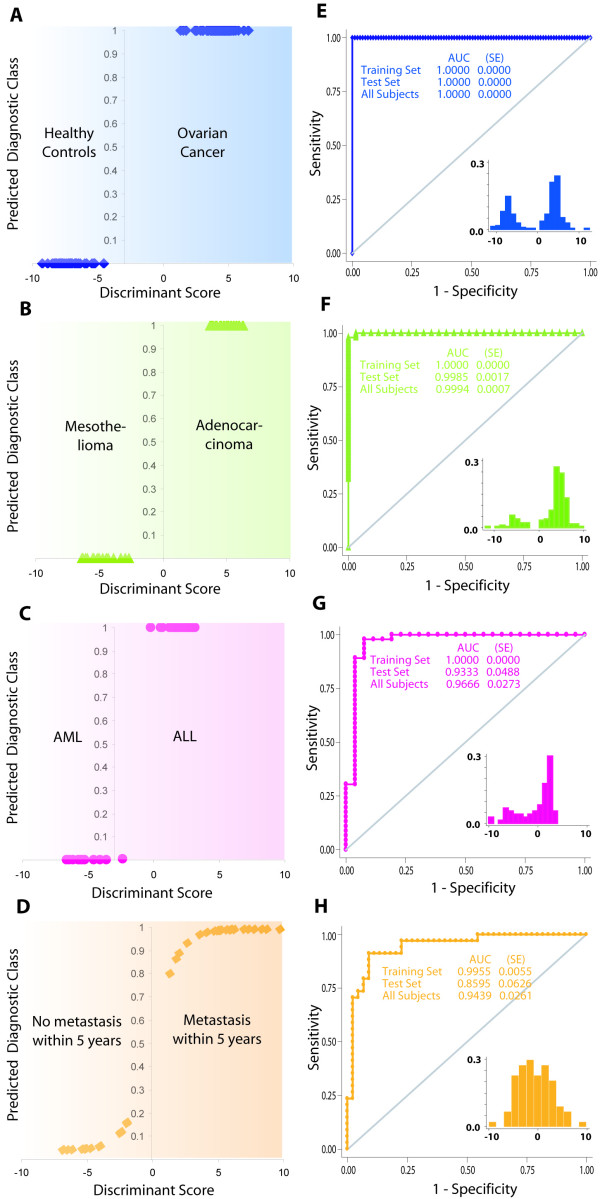
**The diagnostic performance of the proposed statistical algorithm**. (A-D) The probability of the predicted diagnostic class in the training set of each dataset studied. Gradient background indicates a continually increasing or decreasing likelihood of the diagnostic classes. The abscissa indicates the discriminant score generated using the proposed algorithm. (E-H) Evaluation of the diagnostic performance of the proposed algorithm. The plots are ROC curves for the entire dataset (that is training and test sets combined) since the diagnostic performance of the discriminant score was consistently high in the training and test subsets when assessed separately. Area under the ROC curve (AUC) was non-parametrically estimated using the Wilcoxon method. Insets show the strikingly bimodal distribution of the discriminant scores in the entire (that is training and test subsets combined) datasets. SE, standard error.

Table 2 summarizes the results of the linear discriminant analyses. The discriminant scores for each dataset were obtained from the respective training subsets. It was observed that the discriminant score was clearly differentially clustered in the two diagnostic classes in each dataset (Fig. 3A to 3D) and that there was an extremely low or absent misclassification in the training set. The discriminant model fits as indicated by the model R^2^, Mahalanobis D^2^, χ^2^, canonical correlation coefficient, eigenvalues and Wilk's λ (Table 2) demonstrated that the model was highly suited for all the datasets used in this study. Within the training sets the discriminant rule correctly classified 100% of the subjects in the training subsets of all the datasets.

**Table 2:** Summary of the discriminant model performance in the training subsets of the datasets used in the present study

**Statistic**	***Dataset***			
	
	***OvCa ***(n = 132)	***LuMe ***(n = 32)	***LLML ***(n = 38)	**BrCa **(n = 43)
Model R^2^	0.9680	0.9618	0.9170	*0.7436*
Wilk's λ	0.0320	0.0382	0.0830	*0.2564*
Mahanalobis D^2^	127.69	94.38	50.91	*11.57*
χ^2^	416.54	89.77	85.88	*52.40*
Canonical correlation	0.9839	0.9807	0.9576	*0.8623*
Eigenvalue	30.26	25.17	11.05	*2.90*

### Validation of the proposed algorithm

Next, we validated the diagnostic performance of the diagnostic rule in the three steps. First, an examination of the distribution of the discriminant score (insets to Fig 3E to 3H) revealed a markedly bimodal disposition in all the datasets suggesting the likelihood of an efficacious classification.

Second, we generated receiver operating characteristic (ROC) curves using the discriminant score as a predictor of the diagnostic class in the training subset as well as the test subset within each dataset. We observed that the Wilcoxon estimates AUC were high and comparably similar across the training- and test- sets within each of the three datasets (Fig 3E to 3H). Therefore, we estimated the overall prediction accuracy of the discriminant score for each dataset by combining the training- and test-subsets (Fig 3E to 3H).

The AUC for the discriminant score in the training and test sets combined were 100% (95% confidence interval not estimable) for the OvCa dataset, 99.9% (95% confidence interval 99.7%–100%) for the LuMe dataset, 96.7% (95% confidence interval 91.3% – 100%) for the LLML dataset and 94.3% (95 confidence interval 89.3% – 99.5%) for the BrCa dataset. The use of the AUC is only appropriate for this study because the high values of AUC indicate very high partial AUCs related to small false positives, which are required for a study of early detection of cancer. We have further addressed the issue of using AUC in the Discussion section. From these ROC curves we also observed that the cut-off points that maximized the discriminatory performance were -1.09 for the OvCa dataset, 0.43 for the LuMe dataset, -0.26 for the LLML dataset and 0.22 for the BrCa dataset corroborating the general assumption that positive and negative discriminant scores will indicate the two diagnostic classes. At these best cut-off points, we estimated the sensitivity and specificity of the discriminant score, the estimates for sensitivity and specificity were 100% and 100% for the OvCa dataset, 100% and 97% for the LuMe dataset, 98% and 92% for the LLML dataset, and 91% and 91% for the BrCa dataset, respectively.

Third, we scrutinized the list of biomarkers retained in the final model of the synthetically designed dataset (Syn1) of gene expression profile of 1000 hypothetical genes across 2 diagnostic classes in 100 subjects. In truth, the synthetic dataset contained 240 genes that were differentially expressed across the diagnostic classes. For details of the correlation among genes see Section 4 of the additional file 1. We observed that all the 22 genes retained in the final model (see Section 2e in the additional file 1) were included in the list of the known list of differentially expressed genes in this dataset thereby indicating that the algorithm did not falsely discover a differential expression of any gene in this dataset.

### Comparison of the proposed algorithm with other methods

Our algorithm needs a single pass through the dataset for estimating the area under ROC curve for each biomarker and is, therefore, computationally less intense than other data mining techniques used in similar situations e.g. principal components analyses [25,26], singular value decomposition (SVD) [27,28], genetic algorithms (GA) [19], k-nearest neighborhood (kNN) [29,30], support vector machines (SVM) [31-34], logical analysis of data (LAD) [35], classical and fuzzy neural networks (NN) [36-39], self-organizing maps (SOM) [40] and statgram (SG) [41]. A comparison of the results of these techniques with the results from our analyses in the real datasets used in this study (Table 3) demonstrates that the diagnostic accuracy of the proposed algorithm is comparable to or higher than that of the presently available analytical options. Also, in the Syn1 dataset we compared the classification performance of several currently used methods of classification with that of our proposed algorithm (Table 4) and observed a clear advantage of our algorithm – a parsimonious choice of the number of biomarkers without loss of diagnostic performance.

**Table 3:** Comparison of the results of the proposed algorithm with other approaches reported previously using the same datasets

**Dataset**	**Method**	**Reference**	***Accuracy***
OvCa	Principal components	Lilien et al, 2003 [25]	*100*
	Wilcoxon test	Sorace et al, 2003 [53]	*100*
	Logical analysis of data	Alexe et al, 2004 [35]	*100*
	Statgram	Zhu et al, 2003 [41]	*100*
	Genetic algorithm	Petricoin et al, 2002 [19]	*98*
	Proposed algorithm	Present study	*100*
LuMe	Gene expression ratios	Gordon et al, 2002 [18]	*99*
	Proposed algorithm	Present study	*100*
LLML	Self-organizing maps	Toronen et al, 1999 [40]	*90*
	Neural networks	Bicciato et al, 2003 [38]	*97*
	ICED	Bijlani et al, 2003 [65]	*98*
	Support vector machines	Furey et al, 2000 [66]	*88 – 94*
	Proposed algorithm	Present study	*97*
BrCa	Correlation	van't Veer et al [20]	*83*
	Proposed algorithm	Present study	*94*

**Table 4:** Comparison of the results of the proposed algorithm with other approaches using a simulated dataset (Syn1) of 100 samples and 1000 genes

**Method**	**Software**	**Reference**	**# biomarkers used**	***Accuracy (%)***
KMC	Cleaver 1.0	[67]	1000	*89*
kNN	GeneCluster 2.0	[68]	127	*95*
WV	GeneCluster 2.0	[68]	127	*97*
SAM	SAM for Excel	[69]	203	*100*
PAM	PAM for Excel	[69]	224	*100*
SVM	GEPAS	[70]	250	*100*
Proposed	Stata 7.0	[71]	22	*100*

## Discussion

Our proposed statistical algorithm highly accurately classified subjects in all the datasets used in this study. The algorithm is simple, uses statistical techniques that are established in biostatistical literature and can be implemented by most statistical software packages. The choice between parametric versus non-parametric methods for analysis of microarray data has been a matter of intense debate [42]. It is possible that the high diagnostic accuracy achieved by our algorithm may be due to a combination of non-parametric and parametric methods. The suggested approach can be discounted on the basis of the fact that it differs from a more conventional approach of using Student's t test only with regards to the use of area under the ROC curve. It is recognized that the area under the ROC curve follows a Mann-Whitney distribution [43]. Therefore, the first step in the suggested approach can be viewed narrowly as just a non-parametric alternative to the conventional approach.

However, in addition to its advantages mentioned earlier, the proposed algorithm offers one more subtle improvement. Since the true association of a biomarker with the disease remains unknown, various methods exist that indirectly estimate the local false discovery rate (FDR) which gives the probability that a biomarker identified to be associated with disease is in fact falsely identified [44]. Our suggested approach can directly estimate the local FDR (which in the lexicon of diagnostic test performance evaluation can be equated to the inverse of the positive predictive value) of a biomarker by varying the cut-off value for expression level over the observed range of values. This approach also does not presuppose a predefined single estimate of the local FDR. Indeed, the FDR can be allowed to vary based on the definition of expression level used for diagnostic classification. In spite of all these advantages, however, several caveats need to be mentioned before generalizing the results of the present study.

### Study limitation 1: the choice of AUC for ranking biomarkers

The first step of our proposed algorithm makes use of ROC curves and therefore places some restrictions on the generalization of the approach to various situations. The real datasets that we used for validation in our study had diverse aims. For instance, the OvCa dataset was primarily designed for early detection of cancer, the LuMe and LLML datasets have therapeutic implications while the BrCa dataset has a prognostic significance. In the case of early detection of cancer, the emphasis is really on reducing the false positivity rate and, therefore, the area under the entire ROC curve may not be as useful [45]. However, in the analyses conducted in the present study our focus was on discriminating between two classes and all the datasets were used as examples of two class biomarker datasets. Fortuitously, in the OvCa dataset we found a perfect discrimination including the leftward of the ROC curve where the specificity was high.

### Study limitation 2: the proposed algorithm is suitable for two classes only

First, our analysis demonstrates the use of the algorithm only in the situation of dichotomous classification. Theoretically, the technique can be extended to diagnostic outcomes with multiple classes. For example, ROC curves can be drawn for multiple category outcomes and the multi-class counterpart of the discriminant function analysis is available [46-51]. In that case, in the first step of our algorithm one would need to estimate the volume under the multidimensional ROC surface as a non-parametric measure of the diagnostic performance of each biomarker. A potential difficulty can arise in the coding scheme to be used for the outcome variable for multiple linear regression analysis to be conducted in the second step of the algorithm. The coding scheme needs to be compatible with the assumption of linearity implicit in the regression analysis. As this compatibility is unlikely to be known *a priori*, we suggest that the numerical codes for the diagnostic classes be permuted and the regression be used for each permutation. One can, then, choose the regression that gives the best fit. Finally, one can use the multiclass discriminant function analysis on the chosen subset of biomarkers. In the present study, we did not conduct these analyses because Stata modules for estimating volume under surface and for multi-class discriminant analyses are not currently available.

### Study limitation 3: the proposed algorithm can not identify all the differentially expressed biomarkers

The algorithm is designed to make use of the diagnostic performance of a biomarker battery – it is not designed to identify all the biomarkers that are differentially expressed across the diagnostic classes. Consequently, exclusion of a biomarker from those retained in the analysis will not equate to a non-association of that biomarker with the disease state or process. For example, in the Syn1 dataset the algorithm chose only 22 of the actually 240 differentially expressed gene. Also, of the 18 biomarkers in the OvCa dataset that Zhu et al [41] had previously identified, only two were included in our list of 18 independent biomarkers. It has already been pointed out by Baggerly et al [52] and Sorace and Zhan [53] that the reproducibility of different methods in terms of the identified set of biomarkers using the same biomarker dataset is low. Ransohoff argues [54,55] that chance and bias are two major threats to the validity of inferences in studies attempting to associate molecular markers to disease status. For instance, the set of biomarkers identified to be significant may simply be a result of the way the training set was sampled from the full dataset. Indeed, Michiels et al [56,57] have demonstrated that the results obtained in the microarray experiments can be extremely sensitive to the sample size and procedure of selection of the training set. Notably, the training set used by Zhu et al [41] was not the same as the one we used in our analysis.

### Study limitation 4: the choice of retention criterion for stepwise regression

The number of biomarkers finally retained in the stepwise regression analyses, will depend on the criterion used for retention (see Fig A2 in the additional file 1). In stepwise regression models, it is customary to use a probability criterion of 0.2. If this criterion is used in the biomarker analysis, it is evident that more number of biomarkers will be retained in the final model at the cost of an increased complexity of the subsequent discriminant rule. Moreover, considering the high level of accuracy that we obtained even with a very few biomarkers using a retention criterion 20 times as stringent, shows that there is indeed a very small scope for improving the diagnostic performance by relaxing the retention criterion. Again, because the purpose of the analysis was to optimize the diagnostic performance of a subset of biomarkers, to maintain the parsimony of the final regression model, we used a retention criterion of 0.01.

### Study limitation 5: potential bias implicit within the proposed algorithm

We also considered if the algorithm itself is biased in favor of detecting non-existing i.e. false associations of biomarkers with disease states. For this purpose, we generated additional 1000 synthetic datasets (Syn3 – Syn1002) with 100 subjects and 100 biomarkers. Within each of these datasets, each biomarker expression followed a standard normal distribution N(0,1) and was thus non-differentially distributed across diagnostic classes. We then conducted the analyses using the proposed algorithm in each of the 1000 datasets and observed that in 92.8% of these samples the algorithm did not (as expected) find an association between any biomarker and the disease state. In the remaining 72 samples, the algorithm found association of one (67 samples) or two (5 samples) biomarkers with the disease. However, the R^2 ^values indicating the fit of the discriminant function model ranged from 0.0192 – 0.1574 (see Fig A3 in the additional file 1) suggesting that the model fits in these situations of non-existing associations were poor. If one compares these R^2 ^values with those reported in Table 2, it is clear that the algorithm did not detect false associations.

### Issues regarding inferences about validity

To this end, we conducted a further series of analyses to ensure that the diagnostic accuracy of the proposed algorithm was not merely a result of chance or bias. (i) In the OvCa dataset, we generated 100 random training sets of varying sizes and within each of these training sets we estimated the AUC for each of the 15,154 markers (for details, see Fig A4 in the additional file 1). We then assessed the consistency of the AUC estimates in two ways: first, we conducted a factor analysis on the AUC estimates across all samples to assess whether the different training sets map onto a single domain versus multiple domains. We observed that training sets of sizes exceeding 100 were characteristically very similar to each other and consistently identified the same biomarkers. Second, we estimated the Spearman correlation coefficients between each of the random training sets and the training set that we used for the OvCa dataset. Predictably, we again observed that training sets exceeding sizes of 100 were very highly correlated with the training set that we used in this study. Since our training subset comprised of 132 subjects in the OvCa dataset, we believe that our results are unlikely to have been influenced by chance. (ii) We obtained bootstrap estimates of the AUC for all the biomarkers selected in the final model in 500 replicate samples (See Fig A4 in the additional file 1) for all the datasets. We observed that the estimate of AUC that we obtained in the chosen training set was always very close to the mean AUC obtained from 500 replicates even after correcting for sampling bias (see Fig A4C in the additional file 1). Thus, we believe that our results are robust and faithfully reflect true associations between biomarkers and disease status. (iii) Another important threat to validity that we considered was bias. Ransohoff [54] and Baggerly et al [52] also state that if bias in selecting the subjects in the main dataset is hard-wired into the study then demonstration of reproducibility will not be able to address this issue. Therefore, we conducted all our analyses across four different datasets of totally different characteristics. The fact that the proposed algorithm performed very well in all the datasets pointed towards the possibility that it is relatively insensitive to the element of bias specific to each of the chosen datasets. However, it is also possible that all the chosen datasets had negligible bias and therefore our results showed a consistently high performance of the proposed algorithm.

## Conclusion

Within the constraints imposed by the caveats mentioned earlier, our analytical approach demonstrates a technique to translate molecular biomarker data into clinically meaningful and diagnostically useful information. Conceptually and in a broader context, the actual use of the biomarker batteries for detection of the disease status will depend on a conflation of three requisites – existence and severity of the disease under investigation; presence of sensitive and specific biomarkers and the choice of proper statistical analytical methods to ferret out the true relationships between disease and biomarkers. Using real and simulated datasets, in the present study we addressed the last two aspects only. In spite of the high discriminatory performance of the proposed statistical algorithm in the analytical situations considered, caution will be required before recommending molecular biomarkers as diagnostic tests against cancer as the existence and severity of cancer can substantially limit the generalizability of the results. Nevertheless, early and reliable diagnosis is the cornerstone of management of chronic diseases. In that vein and towards that goal, the proposed approach provides a simple, novel and accurate step.

## Methods

### The algorithm

Contingent upon the assumption that each biomarker can be considered as a diagnostic test, we designed a three-step procedure (Fig. 1) to arrive at an optimum combination of the biomarkers that will have a high degree of diagnostic accuracy. A theoretical basis for this improved diagnostic accuracy is that our proposed algorithm identifies a set of independent biomarkers that predict the disease class, and since each member of this set is ensured to be a good predictor of the disease status, a combination of these independent biomarkers will lead to an improved diagnostic accuracy. Following account describe the three steps in our proposed algorithm.

#### Step 1: quantification of diagnostic performance of biomarkers

In the first step, we estimate the diagnostic accuracy of each biomarker by plotting a ROC curve and estimating the area (A) under the ROC curve. The area under an ROC curve captures the overall diagnostic accuracy of the test. In our proposed algorithm, a non-parametric estimate of the area using trapezoidal rule is obtained in this step. Since, the area under an ROC curve is constrained within the interval (0, 1) and since an area of 0.5 represents maximum diagnostic uncertainty [58], we define a diagnostic performance index of the i^th ^biomarker as P_i _= |A_i_-0.5| where A_i _is the estimated area under the ROC curve based on the expression pattern of the i^th ^biomarker. This transformation of the area under ROC curve permits consideration of a bi-directional association of the biomarkers with the disease states such that either over- or under-expression of a particular biomarker can characterize the disease. Thus, the biomarkers can be ranked from highest to lowest values of P_i _(for which the theoretical bounds are 0 and 0.5) with high values indicating diagnostically informative biomarkers and low values suggesting a low discriminatory performance of the biomarkers. However, since the diagnostic performance of one biomarker may be correlated with that of others, choosing the biomarkers with highest P_i _values may not ensure independent and additive contribution of the biomarkers.

#### Step 2: optimizing the number of biomarkers

Therefore, the second step needs to be undertaken to optimize the set of biomarkers with high and independent diagnostic information content in a multivariate setting. Three critical issues need to be considered in this step: choosing an appropriate statistical method for multivariate analysis, choosing the number of diagnostically informative biomarkers to be entered into the multivariate model and using an appropriate method to optimize the number of finally selected biomarkers. With regard to choosing the statistical method, several choices present themselves. An obvious choice is to use the available methods to combine multiple diagnostic tests [16]. However, because one would have only achieved, in step 1, a ranked list of biomarkers; there remains the circular problem of trying to predefine the number of biomarkers to be combined. Another option is to use multivariate regression analyses. As the outcome variable is – by definition – dichotomous, the likely choices can be methods like logistic regression or probit regression. However, since the predictor biomarkers will be selected on the basis of high diagnostic performance (P_i_) it is extremely likely that these will be highly discriminant across the outcome states. Consequently, the regression coefficients in a logistic regression model may not be estimable (because of, for instance, an infinite odds ratio) and these highly informative biomarkers are likely to be automatically dropped from the model by statistical software packages. Therefore, we suggest the use of multivariate linear regression with the outcome variable being the codes for the dichotomous disease states (0/1).

The second issue relates to the number of biomarkers to be included in the multivariate linear regression model. This is not difficult in the context of a multivariate regression model because for a regression model to be identifiable the number of covariates must be less than the number of observations. Thus, the number of most discriminant biomarkers that can be included in this model must be less than the number of subjects included in the analysis. Finally, the third critical issue relates to the method of optimizing the number of biomarkers included in the multivariate analysis. We suggest the approach of a stepwise regression using backward elimination procedure. Considering the fact that the purpose of this step is to optimize the cardinality of the subset of diagnostically useful biomarkers, we suggest the use of a strict retention criterion, that is, the maximum significance value needed to retain a biomarker in the multivariate model. It is evident that the more rigid this criterion, the lesser the number of biomarkers that will be selected in the final model. In our analyses we used a retention criterion of 0.01.

#### Step 3: generating a diagnostic classification based on the optimized subset of biomarkers

Linear discriminant function model is an extension of the linear regression model but can also be used in place of logistic regression [59,60]. Therefore, we suggest the use of a linear discriminant score using the optimized subset of biomarkers identified in the previous step. The discriminant model fit can be assessed by model R^2 ^and its complement – Wilk's λ (which varies between 0 and 1 with a smaller value indicating a better fit). If the model fit is good, then for a given subject a discriminant score can be generated using the unstandardized discriminant regression coefficients and the expression levels of chosen biomarkers. The discriminant function analysis reports the centroids of the discriminant scores for subjects belonging to different diseases states. These can be used to predict the disease status in a given subject. Generally, positive and negative values of the diagnostic score will indicate two different disease states depending on the discriminatory performance of the score. Thus, while use of discriminant function analysis itself is not new to the field of microarray data analysis [61-63], we here propose a novel way to choose a diagnostically discriminant subset of biomarkers by using the results of a stepwise regression analysis procedure.

### Validation of the proposed algorithm

To validate the proposed analytical approach, we used four publicly available datasets (Table 1). Before applying the algorithm to derive a discriminatory rule, we split each original dataset into a training set (to derive the rule) and a validation set (to test whether the rule discriminates between the same diagnostic classes in an independent group of subjects). This split was done once, before any analysis, and the resulting validation set was used only once for the primary analysis. We conducted the validation of this rule in three steps. First, we applied the discriminant rule to the independent test subset. Additionally, we also reported the validation performance of the discriminant score in the training as well as entire dataset. The diagnostic performance of the discriminant score was assessed by estimating sensitivity, specificity and diagnostic accuracy by plotting the ROC curves. Second, we compared the results of our proposed approach to those reported for other available methods of classification using the same four real datasets that we included in the present study. Third, we created a synthetically designed dataset with known set of differentially expressed genes and assessed whether the biomarkers retained in the final model of the proposed algorithm are a subset of the known differentially expressed genes in the synthetic dataset. Also, in this synthetic dataset, we compared the diagnostic performance of the proposed algorithm with that of other methods used for this purpose.

### Datasets

For validation of the proposed algorithm, we used four real datasets and a synthetically designed dataset. The four real datasets (Table 1) were: serum proteomic profile of ovarian cancer subjects and controls (referred here as OvCa data) [19], gene expression data of subjects with adenocarcinoma of lung compared to subjects with mesothelioma (LuMe dataset) [18] and gene expression pattern of subjects with acute lymphocytic leukemia compared with that of subjects with acute myeloid leukemia (LLML dataset) [17] and breast cancer subjects followed prospectively for development of metastasis within or after 5 years of follow-up (BrCa dataset) [20]. The synthetically designed dataset (Syn1) was generated using the SIMAGE software package [64]. This dataset contained expression values for 1000 genes for two diagnostic classes with 50 subjects each. The detailed specifications and input parameters used to generate this dataset are provided in Section 4 in the additional file (see additional file 1). Apart from these five datasets we used a synthetic dataset (Syn2) to examine the influence of data preprocessing on the estimates of AUC (see Section 2 of the additional file 1) and 1000 datasets (Syn3 – Syn1002, see Fig A3 in the additional file 1) to assess the likelihood of false discovery in the use of the proposed algorithm. All the statistical analyses were conducted using Stata 7.0 (College Station, Texas) software package.

## Competing interests

The author(s) declare that they have no competing interests.

## Authors' contributions

MRM and HK conceptualized the study, conducted data analysis and wrote initial and revised drafts of the manuscript. TPT wrote parts of the manuscript and critically reviewed all the drafts. MYK, MAA, YVK and APA critically reviewed the manuscript. All authors read and approved the final manuscript.

## Supplementary Material

Additional File 1 This file contains 4 sections and 4 figures. The first section provides a detailed account of the implementation of the proposed algorithm using Stat 7.0 statistical package. Section 2 demonstrates that the AUC estimates obtained in the first step of the proposed algorithm are not influenced by preprocessing of the data. Section 3 provides detailed output from Stata to support the results described in the text while Section 4 details the input parameters used to generate the Syn1 synthetic dataset. The file also provides ROC curves for individual biomarkers (Fig A1), influence of the retention criterion on the diagnostic performance of the algorithm (Fig A2), distribution of R2 estimates in 1000 synthetic datasets (Fig A3) and influence of the procedure to select a training set on the diagnostic performance of the algorithm (Fig A4). Click here for file
