# Supplementary material for: A simple method to combine multiple molecular biomarkers for dichotomous diagnostic classification
Source: BMC Bioinformatics. 2006 Oct 10;7:442. doi: 10.1186/1471-2105-7-442 (PMC1618410; doi:10.1186/1471-2105-7-442)
Supplement: Additional File 1 — This file contains 4 sections and 4 figures. The first section provides a detailed account of the implementation of the proposed algorithm using Stat 7.0 statistical package. Section 2 demonstrates that the AUC estimates obtained in the first step of the proposed algorithm are not influenced by preprocessing of the data. Section 3 provides detailed output from Stata to support the results described in the text while Section 4 details the input parameters used to generate the Syn1 synthetic dataset. The file also provides ROC curves for individual biomarkers (Fig A1), influence of the retention criterion on the diagnostic performance of the algorithm (Fig A2), distribution of R2 estimates in 1000 synthetic datasets (Fig A3) and influence of the procedure to select a training set on the diagnostic performance of the algorithm (Fig A4). [file 1471-2105-7-442-S1.doc]

**Additional File for:**

**A simple method to combine multiple molecular biomarkers for dichotomous diagnostic classification**

**Manju R. Mamtani^1^, Tushar P. Thakre^1,2^, Mrunal Y. Kalkonde^1^, Manik A Amin^1^, Yogeshwar V. Kalkonde^1^, Amit P. Amin^1^, Hemant R. Kulkarni^1^**

^1^Lata Medical Research Foundation, Nagpur, India

^2^University of North Texas Health Science Center, Fort Worth, Texas, USA

Email addresses:

MRM: [mamtani@uthscsa.edu](mailto:mamtani@uthscsa.edu)

TPT: [tthakre@hsc.unt.edu](mailto:tthakre@hsc.unt.edu)

MYK: [mkalkonde@yahoo.com](mailto:mkalkonde@yahoo.com)

MAA: [saiamin@yahoo.com](mailto:manikamin2004@yahoo.com)

YVK: [yvkalkonde@yahoo.com](mailto:yvkalkonde@yahoo.com)

APA: [amit_p_amin@yahoo.com](mailto:amit_p_amin@yahoo.com)

HRK: [hemant_kulkarnius@yahoo.com](mailto:hemant_kulkarnius@yahoo.com)

**Contents**

| **Section** | **Title** | **Pages** |
| --- | --- | --- |
| 1 | Implementation of the proposed algorithm in Stata 7.0 | 2-3 |
| 2 | AUCs are unaffected by data preprocessing | 4-6 |
| 3 | Detailed supporting results | 7-18 |
| 4 | Input parameters used to synthetically generate the Syn1 dataset by SIMAGE software | 19 |
| Fig. A1 | Receiver-operating characteristic curve for individual biomarkers retained in the final model in step 2 of the algorithm | 20-35 |
| Fig. A2 | Influence of the retention criterion used in stepwise regression analysis | 36-37 |
| Fig. A3 | Distribution of model-fit R^2^ values in the 72 samples | 38 |
| Fig. A4 | Influence of training set selection on the estimates of area under the ROC curve | 39-40 |

Section 1: Implementation of the proposed algorithm in Stata 7.0

All the analysis conducted in the present study were conducted in Stata 7.0 (Stata Corp, College Station, TX) statistical package. While the use of the Stata commands employed in our algorithm is relatively straightforward, for the purposes of clarity of explanations and replication of our results by other investigators, we here provide detailed description of the way in which we conducted our analyses of the microarray dataset using the proposed algorithm.

**Data files and layout**

For each microarray dataset analyzed in the present study, we created three files: first containing all the samples (subjects), the second containing training subset only and third containing the test subset only. It is customary to represent the data from microarray experiments as a genes x samples matrix. To ease statistical analysis in Stata environment, in each of the three files mentioned above we transposed this matrix and represented the data as samples x genes matrix. For transposing the dataset, we used the Stata command xpose. This creates a list of all the genes labeled by an identifier v followed by the index number for the gene. For example, after using the xpose command the 185^th^ row representing the corresponding gene and its expression in all the samples in the original dataset gets transposed to 185^th^ column titled v185 and contains a vertical vector of the expression values in each sample. We then added a variable (as a column in the transposed matrix) titled *status* which represented the diagnostic class for each sample coded as 0 or 1.

**Estimation of area under the receiver-operating characteristic curve for each gene**

As explained in the main text, this analysis was restricted to the training subsets only. For this purpose, we made use of the Stata command roctab. However, since this command only permits the assessment of one predictor at a time, we wrote a Stata program to run this command iteratively for each biomarker and store the results in a separate file. In this file we stored the biomarker id and its performance index (PI) as described in the main text. The code for the Stata program that we used was as follows:

program define calcauc

tempname flnm

display "Estimating Area under ROC for each gene"

postfile `flnm' gene auc using brtrain, replace

forvalues p = 1/24481 {

quietly {

local pn = `"v"'+ string(`p')

roctab status `pn' if test==0

local ta = abs(r(area)-0.5)

post `flnm' (`p') (`ta')

}

}

postclose `flnm'

display "done"

end

We then sorted the biomarkers in descending order of their PI using the Stata command gsort. From this sorted list, we chose the top n-1 biomarkers for further analyses.

**Choosing a subset of biomarkers**

This analysis was also conducted on the training subsets only. We first used the Stata command sw regress for conducting these analyses. In all the stepwise regression analyses, we chose a probability criterion of 0.01 for retaining a biomarker. Thereafter, we used the discrim command to implement discriminant function analyses. From the results of these analyses, we generated a variable titled *score* which was the linear combination of the biomarker expression values and the unstandardized discriminant scores provided by the results of the discrim command. All the model fit indices were then examined for the goodness of the discriminant model fit (Table 2). The discrim command also outputs a graphical display of the class separation (Figure 3, A to D).

**Validation of the proposed algorithm**

This analysis was carried out separately for training and test sets as well as for all the subjects combined. The analysis included generating a ROC curve using score as a predictor of the class, determining the point and interval estimates of the area under the ROC curve and graphically assessing the distribution of the scores for bimodality (Figure 3, E to H). For these analyses also, we used the Stata command roctab.

Section 2: AUCs are unaffected by data preprocessing

**Supporting Results 4. Area under ROC curve for 100 biomarkers using the synthetically generated dataset (Syn2).** First column used transformed (normalized) data while the second column used the raw (untranformed) data. The AUC estimates were not affaected by transformation.

| Marker | AUC transformed | AUC untransformed |
| --- | --- | --- |
| 1 | 0.0504 | 0.0504 |
| 2 | 0.0580 | 0.0580 |
| 3 | 0.0132 | 0.0132 |
| 4 | 0.0288 | 0.0288 |
| 5 | 0.1024 | 0.1024 |
| 6 | 0.0416 | 0.0416 |
| 7 | 0.0052 | 0.0052 |
| 8 | 0.1052 | 0.1052 |
| 9 | 0.0352 | 0.0352 |
| 10 | 0.0596 | 0.0596 |
| 11 | 0.1144 | 0.1144 |
| 12 | 0.0404 | 0.0404 |
| 13 | 0.0048 | 0.0048 |
| 14 | 0.0032 | 0.0032 |
| 15 | 0.0848 | 0.0848 |
| 16 | 0.0276 | 0.0276 |
| 17 | 0.0540 | 0.0540 |
| 18 | 0.0152 | 0.0152 |
| 19 | 0.0520 | 0.0520 |
| 20 | 0.0136 | 0.0136 |
| 21 | 0.0276 | 0.0276 |
| 22 | 0.0756 | 0.0756 |
| 23 | 0.0232 | 0.0232 |
| 24 | 0.0740 | 0.0740 |
| 25 | 0.0468 | 0.0468 |
| 26 | 0.0308 | 0.0308 |
| 27 | 0.0900 | 0.0900 |
| 28 | 0.0204 | 0.0204 |
| 29 | 0.0012 | 0.0012 |
| 30 | 0.0216 | 0.0216 |
| 31 | 0.1012 | 0.1012 |
| 32 | 0.0472 | 0.0472 |
| 33 | 0.0196 | 0.0196 |
| 34 | 0.1340 | 0.1340 |
| 35 | 0.0596 | 0.0596 |
| 36 | 0.0116 | 0.0116 |
| 37 | 0.0260 | 0.0260 |
| 38 | 0.1000 | 0.1000 |
| 39 | 0.0336 | 0.0336 |
| 40 | 0.0332 | 0.0332 |
| 41 | 0.0144 | 0.0144 |
| 42 | 0.0148 | 0.0148 |
| 43 | 0.0604 | 0.0604 |
| 44 | 0.0136 | 0.0136 |
| 45 | 0.0648 | 0.0648 |
| 46 | 0.0188 | 0.0188 |
| 47 | 0.0200 | 0.0200 |
| 48 | 0.0256 | 0.0256 |
| 49 | 0.0488 | 0.0488 |
| 50 | 0.0040 | 0.0040 |
| 51 | 0.0024 | 0.0024 |
| 52 | 0.0384 | 0.0384 |
| 53 | 0.0956 | 0.0956 |
| 54 | 0.1716 | 0.1716 |
| 55 | 0.0300 | 0.0300 |
| 56 | 0.0656 | 0.0656 |
| 57 | 0.0644 | 0.0644 |
| 58 | 0.0580 | 0.0580 |
| 59 | 0.0044 | 0.0044 |
| 60 | 0.1128 | 0.1128 |
| 61 | 0.0508 | 0.0508 |
| 62 | 0.0332 | 0.0332 |
| 63 | 0.0472 | 0.0472 |
| 64 | 0.0252 | 0.0252 |
| 65 | 0.0420 | 0.0420 |
| 66 | 0.0808 | 0.0808 |
| 67 | 0.0432 | 0.0432 |
| 68 | 0.0044 | 0.0044 |
| 69 | 0.0100 | 0.0100 |
| 70 | 0.0312 | 0.0312 |
| 71 | 0.0208 | 0.0208 |
| 72 | 0.0868 | 0.0868 |
| 73 | 0.0368 | 0.0368 |
| 74 | 0.0076 | 0.0076 |
| 75 | 0.0224 | 0.0224 |
| 76 | 0.0672 | 0.0672 |
| 77 | 0.0052 | 0.0052 |
| 78 | 0.1264 | 0.1264 |
| 79 | 0.1912 | 0.1912 |
| 80 | 0.0224 | 0.0224 |
| 81 | 0.0020 | 0.0020 |
| 82 | 0.0360 | 0.0360 |
| 83 | 0.0980 | 0.0980 |
| 84 | 0.0248 | 0.0248 |
| 85 | 0.1188 | 0.1188 |
| 86 | 0.0224 | 0.0224 |
| 87 | 0.0448 | 0.0448 |
| 88 | 0.0656 | 0.0656 |
| 89 | 0.0100 | 0.0100 |
| 90 | 0.0440 | 0.0440 |
| 91 | 0.0432 | 0.0432 |
| 92 | 0.0676 | 0.0676 |
| 93 | 0.0636 | 0.0636 |
| 94 | 0.0768 | 0.0768 |
| 95 | 0.0048 | 0.0048 |
| 96 | 0.0672 | 0.0672 |
| 97 | 0.1348 | 0.1348 |
| 98 | 0.0540 | 0.0540 |
| 99 | 0.0984 | 0.0984 |
| 100 | 0.0248 | 0.0248 |

Section 3: Detailed supporting results

Provided below are the detailed results from Stata 7.0 analysis for stepwise regression and discriminant function analysis in each dataset used in the present study. Section 2(a) – 2(d) are for the real datasets and section 2(e) is for the synthetic dataset.

**Section 2(a): Stepwise regression and discriminant functions for the training component of the OvCa dataset (n=132; 83 cases of ovarian cancer and 49 healthy controls)**

. sw regress status v2237 v2238 v1679 v2236 v2239 v1680 v1681 v1678 v1682 v1683 v2240 v1684 v1687 v1686 v2235 v1685 v1688 v2192 v1736 v1689 v1735 v2311 v2193 v1677 v2310 v2312 v2241 v1600 v1737 v1601 v2191 v2234 v2194 v2313 v544 v543 v1599 v2309 v2242 v182 v545 v1594 v1602 v1734 v1738 v1690 v1676 v542 v181 v1598 v1674 v1593 v546 v2195 v2314 v1675 v1603 v2666 v1596 v2665 v2667 v6782 v2190 v1597 v2668 v1604 v1595 v6802 v6803 v547 v2243 v541 v576 v183 v2664 v701 v9608 v567 v575 v9607 v569 v568 v574 v573 v9609 v570 v572 v9606 v1605 v6781 v566 v571 v700 v2308 v9605 v5534 v6783 v1733 v579 v563, pr(0.01)

begin with full model

p = 0.9887 >= 0.0100 removing v1678

p = 0.9812 >= 0.0100 removing v1602

p = 0.9726 >= 0.0100 removing v6803

p = 0.9699 >= 0.0100 removing v1737

p = 0.9717 >= 0.0100 removing v1736

p = 0.8998 >= 0.0100 removing v573

p = 0.8887 >= 0.0100 removing v1685

p = 0.9028 >= 0.0100 removing v544

p = 0.8779 >= 0.0100 removing v2240

p = 0.8832 >= 0.0100 removing v182

p = 0.8051 >= 0.0100 removing v9608

p = 0.8001 >= 0.0100 removing v2666

p = 0.9040 >= 0.0100 removing v2664

p = 0.9569 >= 0.0100 removing v2665

p = 0.7497 >= 0.0100 removing v1682

p = 0.6179 >= 0.0100 removing v2312

p = 0.6671 >= 0.0100 removing v567

p = 0.6097 >= 0.0100 removing v2193

p = 0.5108 >= 0.0100 removing v2314

p = 0.5620 >= 0.0100 removing v2313

p = 0.4742 >= 0.0100 removing v1601

p = 0.4200 >= 0.0100 removing v1738

p = 0.4538 >= 0.0100 removing v5534

p = 0.4335 >= 0.0100 removing v2195

p = 0.3932 >= 0.0100 removing v571

p = 0.3719 >= 0.0100 removing v572

p = 0.3920 >= 0.0100 removing v1679

p = 0.4208 >= 0.0100 removing v547

p = 0.3940 >= 0.0100 removing v1686

p = 0.3155 >= 0.0100 removing v574

p = 0.4114 >= 0.0100 removing v1677

p = 0.5148 >= 0.0100 removing v1675

p = 0.2550 >= 0.0100 removing v579

p = 0.2333 >= 0.0100 removing v1690

p = 0.3704 >= 0.0100 removing v575

p = 0.2902 >= 0.0100 removing v568

p = 0.5132 >= 0.0100 removing v569

p = 0.2183 >= 0.0100 removing v1676

p = 0.1698 >= 0.0100 removing v1596

p = 0.5216 >= 0.0100 removing v1597

p = 0.1761 >= 0.0100 removing v563

p = 0.2323 >= 0.0100 removing v541

p = 0.6756 >= 0.0100 removing v542

p = 0.1163 >= 0.0100 removing v1681

p = 0.2461 >= 0.0100 removing v6802

p = 0.1265 >= 0.0100 removing v2667

p = 0.1429 >= 0.0100 removing v2243

p = 0.4876 >= 0.0100 removing v2242

p = 0.1124 >= 0.0100 removing v9605

p = 0.1164 >= 0.0100 removing v1683

p = 0.1454 >= 0.0100 removing v1684

p = 0.1514 >= 0.0100 removing v1687

p = 0.1223 >= 0.0100 removing v566

p = 0.1826 >= 0.0100 removing v1595

p = 0.1858 >= 0.0100 removing v1593

p = 0.0758 >= 0.0100 removing v181

p = 0.0281 >= 0.0100 removing v2241

p = 0.0354 >= 0.0100 removing v1599

p = 0.4998 >= 0.0100 removing v1600

p = 0.1617 >= 0.0100 removing v1598

p = 0.0781 >= 0.0100 removing v701

p = 0.0718 >= 0.0100 removing v1689

p = 0.0457 >= 0.0100 removing v6781

p = 0.1864 >= 0.0100 removing v6783

p = 0.1489 >= 0.0100 removing v6782

p = 0.0359 >= 0.0100 removing v2234

p = 0.1124 >= 0.0100 removing v2237

p = 0.5008 >= 0.0100 removing v2238

p = 0.0590 >= 0.0100 removing v2239

p = 0.0595 >= 0.0100 removing v2235

p = 0.0761 >= 0.0100 removing v1603

p = 0.2481 >= 0.0100 removing v1604

p = 0.4660 >= 0.0100 removing v1605

p = 0.0636 >= 0.0100 removing v570

p = 0.0366 >= 0.0100 removing v1688

p = 0.0433 >= 0.0100 removing v2190

p = 0.1914 >= 0.0100 removing v1733

p = 0.0769 >= 0.0100 removing v1734

p = 0.5052 >= 0.0100 removing v1735

p = 0.2948 >= 0.0100 removing v2192

p = 0.5264 >= 0.0100 removing v2191

p = 0.0408 >= 0.0100 removing v2194

Source | SS df MS Number of obs = 132

-------------+------------------------------ F( 18, 113) = 189.99

Model | 29.8250967 18 1.65694981 Prob > F = 0.0000

Residual | .985509392 113 .008721322 R-squared = 0.9680

-------------+------------------------------ Adj R-squared = 0.9629

Total | 30.8106061 131 .235195466 Root MSE = .09339

------------------------------------------------------------------------------

status | Coef. Std. Err. t P>|t| [95% Conf. Interval]

-------------+----------------------------------------------------------------

v2668 | -.0033325 .0011428 -2.92 0.004 -.0055967 -.0010683

v2310 | .4016585 .1050694 3.82 0.000 .1934971 .60982

v183 | -1.405806 .1090191 -12.90 0.000 -1.621792 -1.189819

v2236 | .0281942 .0035535 7.93 0.000 .021154 .0352344

v700 | .0084214 .0019957 4.22 0.000 .0044676 .0123752

v1680 | -.015582 .0017125 -9.10 0.000 -.0189749 -.0121892

v9607 | -.4522872 .1177644 -3.84 0.000 -.6855996 -.2189747

v9606 | .2854334 .0748845 3.81 0.000 .1370737 .4337932

v2309 | -.3746895 .0909307 -4.12 0.000 -.5548396 -.1945395

v1674 | -.086489 .0207674 -4.16 0.000 -.1276329 -.045345

v1594 | .1129936 .0197357 5.73 0.000 .0738936 .1520935

v545 | -.0766166 .0130247 -5.88 0.000 -.1024209 -.0508123

v576 | -.2232032 .0315431 -7.08 0.000 -.2856957 -.1607107

v543 | .0250613 .004457 5.62 0.000 .0162312 .0338914

v546 | .0807223 .0134419 6.01 0.000 .0540914 .1073533

v2308 | .1417438 .0312715 4.53 0.000 .0797892 .2036984

v9609 | .1601955 .0457641 3.50 0.001 .0695285 .2508624

v2311 | -.1776602 .058564 -3.03 0.003 -.293686 -.0616344

_cons | 7.025807 .5011152 14.02 0.000 6.033007 8.018606

------------------------------------------------------------------------------

. discrim status v2668 v2310 v183 v2236 v700 v1680 v9607 v9606 v2309 v1674 v1594 v545 v576 v543 v546 v2

> 308 v9609 v2311

Dichotomous Discriminant Analysis

Observations = 132 Obs Group 0 = 49

Indep variables = 18 Obs Group 1 = 83

Centroid 0 = -7.1054 R-square = 0.9680

Centroid 1 = 4.1947 Mahalanobis = 127.6922

Grand Cntd = -2.9106

Eigenvalue = 30.2637 Wilk's Lambda = 0.0320

Canon. Corr. = 0.9839 Chi-square = 416.5374

Eta Squared = 0.9680 Sign Chi2 = 0.0000

Discrim Function Unstandardized

Variable Coefficients Coefficients

-------------------------------------------------

v2668 0.4396 -0.0389

v2310 -52.9834 4.6888

v183 185.4419 -16.4107

v2236 -3.7191 0.3291

v700 -1.1109 0.0983

v1680 2.0554 -0.1819

v9607 59.6619 -5.2798

v9606 -37.6519 3.3320

v2309 49.4259 -4.3739

v1674 11.4089 -1.0096

v1594 -14.9052 1.3190

v545 10.1066 -0.8944

v576 29.4431 -2.6056

v543 -3.3059 0.2926

v546 -10.6482 0.9423

v2308 -18.6976 1.6546

v9609 -21.1316 1.8700

v2311 23.4354 -2.0739

constant -860.2856 74.6755

**Section 2(b): Stepwise regression and discriminant functions for the training component of the LuMe dataset (n=32; 16 cases of lung adenocarcinoma and 16 cases of mesothelioma)**

. sw regress status v1136 v1246 v2039 v2549 v3250 v3844 v5356 v6571 v7249 v7765 v9863 v11015 v11368 v11485 v12114 v12523 v3490 v8005 v8537 v11957 v12254 v541 v718 v2421 v3333 v4336 v5301 v9474 v11841 v12186, pr(0.01)

begin with full model

p = 0.9964 >= 0.0100 removing v7249

p = 0.9838 >= 0.0100 removing v8537

p = 0.9396 >= 0.0100 removing v541

p = 0.8540 >= 0.0100 removing v2421

p = 0.8891 >= 0.0100 removing v8005

p = 0.8051 >= 0.0100 removing v12254

p = 0.7211 >= 0.0100 removing v1246

p = 0.4411 >= 0.0100 removing v11957

p = 0.4112 >= 0.0100 removing v5301

p = 0.4800 >= 0.0100 removing v718

p = 0.3732 >= 0.0100 removing v12114

p = 0.3558 >= 0.0100 removing v3250

p = 0.3111 >= 0.0100 removing v5356

p = 0.3254 >= 0.0100 removing v2039

p = 0.2133 >= 0.0100 removing v7765

p = 0.2165 >= 0.0100 removing v3333

p = 0.1970 >= 0.0100 removing v12523

p = 0.1643 >= 0.0100 removing v9474

p = 0.1613 >= 0.0100 removing v11485

p = 0.0514 >= 0.0100 removing v3844

p = 0.2364 >= 0.0100 removing v3490

p = 0.0977 >= 0.0100 removing v12186

p = 0.1312 >= 0.0100 removing v6571

p = 0.1358 >= 0.0100 removing v11841

p = 0.2743 >= 0.0100 removing v11015

Source | SS df MS Number of obs = 32

-------------+------------------------------ F( 5, 26) = 130.87

Model | 7.69428477 5 1.53885695 Prob > F = 0.0000

Residual | .305715226 26 .011758278 R-squared = 0.9618

-------------+------------------------------ Adj R-squared = 0.9544

Total | 8.00 31 .258064516 Root MSE = .10844

------------------------------------------------------------------------------

status | Coef. Std. Err. t P>|t| [95% Conf. Interval]

-------------+----------------------------------------------------------------

v1136 | .0003008 .0001034 2.91 0.007 .0000882 .0005134

v11368 | .0036792 .0010168 3.62 0.001 .0015892 .0057692

v9863 | -.0001664 .0000551 -3.02 0.006 -.0002798 -.0000531

v2549 | -.0005334 .000101 -5.28 0.000 -.000741 -.0003259

v4336 | -.0001204 .0000343 -3.51 0.002 -.0001909 -.0000498

_cons | .6613909 .1467676 4.51 0.000 .3597058 .9630759

------------------------------------------------------------------------------

. discrim status v1136 v11368 v9863 v2549 v4336

Dichotomous Discriminant Analysis

Observations = 32 Obs Group 0 = 16

Indep variables = 5 Obs Group 1 = 16

Centroid 0 = -4.8575 R-square = 0.9618

Centroid 1 = 4.8575 Mahalanobis = 94.3805

Grand Cntd = 0.0000

Eigenvalue = 25.1681 Wilk's Lambda = 0.0382

Canon. Corr. = 0.9807 Chi-square = 89.7749

Eta Squared = 0.9618 Sign Chi2 = 0.0000

Discrim Function Unstandardized

Variable Coefficients Coefficients

-------------------------------------------------

v1136 -0.0295 0.0030

v11368 -0.3610 0.0372

v9863 0.0163 -0.0017

v2549 0.0523 -0.0054

v4336 0.0118 -0.0012

constant -15.8374 1.6302

**Section 2(c): Stepwise regression and discriminant functions for the training component of the LLML dataset (n=38; 27 cases of acute lymphocytic leukemia and 11 cases of acute myeloid leukemia)**

. sw regress status v4847 v1882 v3320 v6218 v760 v1834 v1745 v2020 v4499 v5039 v2267 v5772 v6041 v2354 v4377 v6855 v2121 v3252 v248 v2015 v312 v1144 v1630 v4535 v6281 v2233 v4107 v4780 v6539 v3847, pr(0.01)

begin with full model

p = 0.9741 >= 0.0100 removing v6041

p = 0.9598 >= 0.0100 removing v1882

p = 0.8282 >= 0.0100 removing v4499

p = 0.8630 >= 0.0100 removing v1630

p = 0.8190 >= 0.0100 removing v4847

p = 0.6478 >= 0.0100 removing v2121

p = 0.6278 >= 0.0100 removing v6281

p = 0.6538 >= 0.0100 removing v2015

p = 0.6482 >= 0.0100 removing v5039

p = 0.5216 >= 0.0100 removing v3252

p = 0.4003 >= 0.0100 removing v4535

p = 0.2500 >= 0.0100 removing v5772

p = 0.4093 >= 0.0100 removing v6855

p = 0.1455 >= 0.0100 removing v6218

p = 0.1881 >= 0.0100 removing v4377

p = 0.1272 >= 0.0100 removing v3847

p = 0.0622 >= 0.0100 removing v2020

p = 0.0724 >= 0.0100 removing v2233

p = 0.0883 >= 0.0100 removing v4780

p = 0.0423 >= 0.0100 removing v2354

p = 0.1315 >= 0.0100 removing v1144

p = 0.1299 >= 0.0100 removing v760

p = 0.1350 >= 0.0100 removing v312

p = 0.0157 >= 0.0100 removing v2267

p = 0.0835 >= 0.0100 removing v3320

p = 0.0207 >= 0.0100 removing v4107

p = 0.0759 >= 0.0100 removing v248

Source | SS df MS Number of obs = 38

-------------+------------------------------ F( 3, 34) = 125.27

Model | 7.16734398 3 2.38911466 Prob > F = 0.0000

Residual | .648445495 34 .019071926 R-squared = 0.9170

-------------+------------------------------ Adj R-squared = 0.9097

Total | 7.81578947 37 .211237553 Root MSE = .1381

------------------------------------------------------------------------------

status | Coef. Std. Err. t P>|t| [95% Conf. Interval]

-------------+----------------------------------------------------------------

v1745 | -.0001386 .0000375 -3.70 0.001 -.0002148 -.0000625

v6539 | -.0002284 .000026 -8.77 0.000 -.0002813 -.0001754

v1834 | -.000649 .000084 -7.73 0.000 -.0008196 -.0004783

_cons | 1.228861 .0349177 35.19 0.000 1.1579 1.299822

------------------------------------------------------------------------------

. discrim status v1745 v6539 v1834

Dichotomous Discriminant Analysis

Observations = 38 Obs Group 0 = 11

Indep variables = 3 Obs Group 1 = 27

Centroid 0 = -5.0698 R-square = 0.9170

Centroid 1 = 2.0655 Mahalanobis = 50.9113

Grand Cntd = -3.0043

Eigenvalue = 11.0531 Wilk's Lambda = 0.0830

Canon. Corr. = 0.9576 Chi-square = 85.8817

Eta Squared = 0.9170 Sign Chi2 = 0.0000

Discrim Function Unstandardized

Variable Coefficients Coefficients

-------------------------------------------------

v1745 0.0077 -0.0011

v6539 0.0127 -0.0018

v1834 0.0360 -0.0050

constant -39.4948 4.0330

**Section 2(d): Stepwise regression and discriminant functions for the training component of the BrCa dataset (n=43; 17 cases that metastasized within 5 years and 26 cases that did not metastasize within 5 years of follow-up)**

. sw regress status v13366 v15326 v10987 v15932 v14696 v10480 v4050 v8531 v15115 v24143 v13902 v8179 v15080 v5821 v7300 v13998 v9059 v15874 v11025 v22068 v5261 v9600 v19400 v785 v22845 v8662 v11000 v15982 v19241 v14950 v9566 v7653 v15525 v11242 v10796 v3240 v3463 v23569 v12479 v14169 v2850, pr(0.01)

begin with full model

p = 0.9981 >= 0.0100 removing v14696

p = 0.9945 >= 0.0100 removing v11025

p = 0.9778 >= 0.0100 removing v19241

p = 0.9009 >= 0.0100 removing v10987

p = 0.5999 >= 0.0100 removing v13902

p = 0.5597 >= 0.0100 removing v785

p = 0.5166 >= 0.0100 removing v10480

p = 0.4442 >= 0.0100 removing v3463

p = 0.5133 >= 0.0100 removing v14169

p = 0.3009 >= 0.0100 removing v8662

p = 0.2148 >= 0.0100 removing v15080

p = 0.4037 >= 0.0100 removing v11242

p = 0.4444 >= 0.0100 removing v14950

p = 0.4224 >= 0.0100 removing v2850

p = 0.3497 >= 0.0100 removing v15115

p = 0.3308 >= 0.0100 removing v19400

p = 0.0685 >= 0.0100 removing v15982

p = 0.0831 >= 0.0100 removing v23569

p = 0.0823 >= 0.0100 removing v11000

p = 0.1801 >= 0.0100 removing v13998

p = 0.0889 >= 0.0100 removing v12479

p = 0.1210 >= 0.0100 removing v8179

p = 0.0811 >= 0.0100 removing v15874

p = 0.1521 >= 0.0100 removing v7653

p = 0.2658 >= 0.0100 removing v8531

p = 0.0513 >= 0.0100 removing v4050

p = 0.0996 >= 0.0100 removing v22845

p = 0.1115 >= 0.0100 removing v10796

p = 0.0696 >= 0.0100 removing v5821

p = 0.1141 >= 0.0100 removing v9600

p = 0.1193 >= 0.0100 removing v9059

p = 0.0940 >= 0.0100 removing v15326

p = 0.1124 >= 0.0100 removing v9566

p = 0.0999 >= 0.0100 removing v3240

p = 0.1598 >= 0.0100 removing v24143

p = 0.0300 >= 0.0100 removing v15525

Source | SS df MS Number of obs = 43

-------------+------------------------------ F( 5, 37) = 21.46

Model | 7.64364258 5 1.52872852 Prob > F = 0.0000

Residual | 2.63542719 37 .071227762 R-squared = 0.7436

-------------+------------------------------ Adj R-squared = 0.7090

Total | 10.2790698 42 .244739756 Root MSE = .26689

------------------------------------------------------------------------------

status | Coef. Std. Err. t P>|t| [95% Conf. Interval]

-------------+----------------------------------------------------------------

v13366 | -2.626918 .728197 -3.61 0.001 -4.102386 -1.151451

v22068 | -3.027666 .6793132 -4.46 0.000 -4.404085 -1.651247

v5261 | 2.337108 .7082376 3.30 0.002 .9020823 3.772134

v15932 | 2.360049 .7515117 3.14 0.003 .8373416 3.882756

v7300 | -2.308767 .5189047 -4.45 0.000 -3.360168 -1.257367

_cons | -.4720346 .1559173 -3.03 0.004 -.7879531 -.1561161

------------------------------------------------------------------------------

. discrim status v13366 v22068 v5261 v15932 v7300

Dichotomous Discriminant Analysis

Observations = 43 Obs Group 0 = 26

Indep variables = 5 Obs Group 1 = 17

Centroid 0 = -1.3447 R-square = 0.7436

Centroid 1 = 2.0566 Mahalanobis = 11.5686

Grand Cntd = 0.7119

Eigenvalue = 2.9003 Wilk's Lambda = 0.2564

Canon. Corr. = 0.8623 Chi-square = 52.4010

Eta Squared = 0.7436 Sign Chi2 = 0.0000

Discrim Function Unstandardized

Variable Coefficients Coefficients

-------------------------------------------------

v13366 40.8676 -12.0154

v22068 47.1022 -13.8484

v5261 -36.3590 10.6899

v15932 -36.7159 10.7948

v7300 35.9181 -10.5602

constant 14.7048 -3.9674

**Section 2(e): Stepwise regression and discriminant functions for the synthetic dataset (Syn1) generated using the Simage software (n=100; 50 samples of diagnostic class 1 and 50 cases of diagnostic class 0)**

. sw regress status v860 v402 v444 v903 v603 v914 v820 v168 v981 v246 v443 v891 v679 v585 v951 v309 v54 v966 v727 v643 v666 v695 v405 v992 v213 v482 v779 v824 v579 v705 v980 v319 v742 v686 v228 v950 v71 v45 v680 v264 v863 v564 v427 v926 v440 v188 v274 v186 v462 v255 v887 v837 v641 v534 v170 v761 v591 v387 v604 v32 v203 v662 v953 v442 v988 v755 v827 v958 v848 v777 v454 v555 v956 v266 v409 v34 v859 v982 v185 v344 v149 v328 v547 v535 v346 v999 v945 v150 v10 v488 v873 v199 v297 v919 v614 v435 v360 v320, pr(0.01)

begin with full model

p = 0.9785 >= 0.0100 removing v988

p = 0.9709 >= 0.0100 removing v45

p = 0.8941 >= 0.0100 removing v387

p = 0.8471 >= 0.0100 removing v951

p = 0.7554 >= 0.0100 removing v761

p = 0.7314 >= 0.0100 removing v779

p = 0.7854 >= 0.0100 removing v488

p = 0.6924 >= 0.0100 removing v344

p = 0.5755 >= 0.0100 removing v462

p = 0.5746 >= 0.0100 removing v742

p = 0.4897 >= 0.0100 removing v54

p = 0.3630 >= 0.0100 removing v185

p = 0.2729 >= 0.0100 removing v149

p = 0.2641 >= 0.0100 removing v547

p = 0.1283 >= 0.0100 removing v914

p = 0.1895 >= 0.0100 removing v454

p = 0.3382 >= 0.0100 removing v555

p = 0.1655 >= 0.0100 removing v170

p = 0.0713 >= 0.0100 removing v695

p = 0.1029 >= 0.0100 removing v309

p = 0.0504 >= 0.0100 removing v992

p = 0.0453 >= 0.0100 removing v982

p = 0.2403 >= 0.0100 removing v409

p = 0.1657 >= 0.0100 removing v666

p = 0.1874 >= 0.0100 removing v443

p = 0.1480 >= 0.0100 removing v860

p = 0.1782 >= 0.0100 removing v966

p = 0.1459 >= 0.0100 removing v641

p = 0.1504 >= 0.0100 removing v999

p = 0.0693 >= 0.0100 removing v188

p = 0.1261 >= 0.0100 removing v686

p = 0.1533 >= 0.0100 removing v777

p = 0.0408 >= 0.0100 removing v727

p = 0.0611 >= 0.0100 removing v213

p = 0.0987 >= 0.0100 removing v444

p = 0.0893 >= 0.0100 removing v919

p = 0.3159 >= 0.0100 removing v34

p = 0.2200 >= 0.0100 removing v679

p = 0.0490 >= 0.0100 removing v264

p = 0.0478 >= 0.0100 removing v402

p = 0.0665 >= 0.0100 removing v297

p = 0.0777 >= 0.0100 removing v274

p = 0.1374 >= 0.0100 removing v534

p = 0.0813 >= 0.0100 removing v482

p = 0.2010 >= 0.0100 removing v266

p = 0.0800 >= 0.0100 removing v203

p = 0.1366 >= 0.0100 removing v956

p = 0.1189 >= 0.0100 removing v662

p = 0.0588 >= 0.0100 removing v168

p = 0.0191 >= 0.0100 removing v255

p = 0.1241 >= 0.0100 removing v981

p = 0.0370 >= 0.0100 removing v228

p = 0.1422 >= 0.0100 removing v32

p = 0.0332 >= 0.0100 removing v863

p = 0.0359 >= 0.0100 removing v535

p = 0.0678 >= 0.0100 removing v246

p = 0.0794 >= 0.0100 removing v10

p = 0.0746 >= 0.0100 removing v891

p = 0.1140 >= 0.0100 removing v953

p = 0.0917 >= 0.0100 removing v950

p = 0.1662 >= 0.0100 removing v958

p = 0.1108 >= 0.0100 removing v319

p = 0.1624 >= 0.0100 removing v945

p = 0.0234 >= 0.0100 removing v320

p = 0.0832 >= 0.0100 removing v926

p = 0.0650 >= 0.0100 removing v824

p = 0.0872 >= 0.0100 removing v328

p = 0.1040 >= 0.0100 removing v71

p = 0.0548 >= 0.0100 removing v346

p = 0.1400 >= 0.0100 removing v680

p = 0.0393 >= 0.0100 removing v848

p = 0.0486 >= 0.0100 removing v591

p = 0.1280 >= 0.0100 removing v579

p = 0.0903 >= 0.0100 removing v405

p = 0.0766 >= 0.0100 removing v427

p = 0.1006 >= 0.0100 removing v837

Source | SS df MS Number of obs = 100

-------------+------------------------------ F( 22, 77) = 200.33

Model | 24.5707106 22 1.11685048 Prob > F = 0.0000

Residual | .429289421 77 .005575187 R-squared = 0.9828

-------------+------------------------------ Adj R-squared = 0.9779

Total | 25.00 99 .252525253 Root MSE = .07467

------------------------------------------------------------------------------

status | Coef. Std. Err. t P>|t| [95% Conf. Interval]

-------------+----------------------------------------------------------------

v435 | -.2620635 .0642337 -4.08 0.000 -.3899692 -.1341577

v604 | -.1645926 .0421342 -3.91 0.000 -.2484924 -.0806928

v442 | -.2337953 .0533199 -4.38 0.000 -.3399689 -.1276218

v903 | .1300395 .0189452 6.86 0.000 .0923148 .1677643

v603 | .153139 .0217831 7.03 0.000 .1097632 .1965147

v360 | -.1858544 .0463513 -4.01 0.000 -.2781516 -.0935573

v820 | -.1801785 .0554173 -3.25 0.002 -.2905283 -.0698286

v199 | .067984 .0210413 3.23 0.002 .0260855 .1098826

v186 | .1699537 .0369051 4.61 0.000 .0964664 .2434411

v859 | .0892156 .0303792 2.94 0.004 .0287229 .1497082

v564 | -.0463239 .01204 -3.85 0.000 -.0702986 -.0223492

v440 | .0716013 .0232678 3.08 0.003 .0252692 .1179334

v705 | .0976142 .0329426 2.96 0.004 .0320171 .1632113

v585 | .0482841 .0138633 3.48 0.001 .0206787 .0758895

v614 | .276348 .0823714 3.35 0.001 .1123255 .4403704

v827 | .0496345 .0178492 2.78 0.007 .0140922 .0851768

v150 | -.0344334 .0073956 -4.66 0.000 -.0491599 -.019707

v980 | -.0857125 .0128774 -6.66 0.000 -.1113547 -.0600703

v755 | .1428502 .0362806 3.94 0.000 .0706064 .215094

v643 | -.1802003 .0229416 -7.85 0.000 -.225883 -.1345176

v887 | -.2217775 .0492205 -4.51 0.000 -.319788 -.123767

v873 | .0948827 .0237341 4.00 0.000 .0476221 .1421433

_cons | .3069672 .0835852 3.67 0.000 .1405279 .4734066

------------------------------------------------------------------------------

. discrim status v435 v604 v442 v903 v603 v360 v820 v199 v186 v859 v564 v440 v705 v585 v614 v827 v150 v

> 980 v755 v643 v887 v873

Dichotomous Discriminant Analysis

Observations = 100 Obs Group 0 = 50

Indep variables = 22 Obs Group 1 = 50

Centroid 0 = -7.4894 R-square = 0.9828

Centroid 1 = 7.4894 Mahalanobis = 224.3642

Grand Cntd = 0.0000

Eigenvalue = 57.2358 Wilk's Lambda = 0.0172

Canon. Corr. = 0.9914 Chi-square = 353.6115

Eta Squared = 0.9828 Sign Chi2 = 0.0000

Discrim Function Unstandardized

Variable Coefficients Coefficients

-------------------------------------------------

v435 59.8250 -3.9940

v604 37.5739 -2.5085

v442 53.3718 -3.5632

v903 -29.6860 1.9819

v603 -34.9592 2.3339

v360 42.4276 -2.8325

v820 41.1319 -2.7460

v199 -15.5197 1.0361

v186 -38.7978 2.5902

v859 -20.3665 1.3597

v564 10.5750 -0.7060

v440 -16.3454 1.0912

v705 -22.2838 1.4877

v585 -11.0225 0.7359

v614 -63.0859 4.2117

v827 -11.3308 0.7565

v150 7.8606 -0.5248

v980 19.5668 -1.3063

v755 -32.6104 2.1771

v643 41.1369 -2.7463

v887 50.6283 -3.3800

v873 -21.6602 1.4461

constant 44.0663 -2.9419

.

Section 4: Input parameters used to synthetically generate the Syn1 dataset by SIMAGE software

**Parameter Description Value**

Array number of grid rows 9

Array number of grid columns 4

Number of spots in a grid row 18

Number of spots in a grid column 18

Number of spot pins 12

Number of technical replicates 1

Number of genes (0 = max) 1000

Number of slides 50

Perform dye swaps no

Gene expression filter yes

Reset gene filter for each slide no

Mean signal 11.492

Change in log2ratio due to upregulation 0.832

Change in log2ratio due to downregulation 0.605

Variance of gene expression 1.775

% of upregulated genes 15

% of downregulated genes 9

Correlation between channels 0.981

Dye filter yes

Reset dye filter for each slide yes

Channel (dye) variation 0.51

Gene x Dye 0

Error filter yes

Reset error filter for each slide yes

Random noise standard deviation 0.219

Tail behaviour in the MA plot 0.09

Non-linearity filter yes

Reset non-linearity filter for each slide yes

Non-linearity parameter curvature 0.025

Non-linearity parameter tilt 0.777

Non-linearity from scanner filter yes

Reset non-linearity scanner filter for each slide yes

Scanning device bias 0.295

spotpin deviation filter yes

Reset spotpin filter for each slide no

spotpin variation 0.36

Background filter yes

Reset background filter for each slide yes

Number of background densities 5

Mean SD per background density 0.3

Maximum of the background signal (%) relative to the non-background 100

SD of the random noise for the background signals 0.1

Background gradient filter yes

Reset gradient filter for each slide yes

Maximum slope of the linear tilt 700

Missing values filter yes

Reset missing spots filter for each slide yes

Number of hairs 10

Maximum length of hair 20

Number of discs 6

Average radius disc 10

Number of missing spots 50

Figure A1**. Receiver-operating characteristic curve for individual biomarkers retained in the final model in step 2 of the algorithm. (A-R) M/Z values for markers in the OvCa dataset, (S-W) Genes in the LuMe dataset, (X-Z) Genes in the LLML dataset, (AA-AE) Genes in the BrCa dataset.**

**(A) M/Z = 2.8549**

**(B) M/Z = 25.4956**

**(C) M/Z = 25.6844**

**(D) M/Z = 25.7791**

**(E) M/Z = 28.7005**

**(F) M/Z = 42.4388**

**(G) M/Z = 220.7513**

**(H) M/Z = 243.4940**

**(I) M/Z = 245.2447**

**(J) M/Z = 434.6859**

**(K) M/Z = 463.1559**

**(L) M/Z = 463.5577**

**(M) M/Z = 463.9596**

**(N) M/Z = 464.3617**

**(O) M/Z = 619.0509**

**(P) M/Z = 8033.385**

**(Q) M/Z = 8035.058**

**(R) M/Z = 8038.405**

**(S) 2047_s_at**

**(T) 41286_at**

**(U) 39795_at**

**(V) 32551-at**

**(W) 34320_at**

**(X) LYN V-yes-1 Yamaguchi sarcoma viral related oncogene homolog**

**(Y) Calpain 2**

**(Z) Epb72 gene**

**(AA) NGFIA-binding protein-2 (NAB2) Gene**

**(AB) Aurora kinase A interacting protein 1 (AURKAIP1) Gene**

**(AC) V-set domain containing T cell activation inhibitor 1 (VTCN1) Gene**

**(AD) Zinc finger protein 473 (ZNF473) Gene**

**(AE) Leucine-rich repeats and calponin homology (CH) domain containing 3 (LRCH3) Gene**

Figure A2**. Influence of the retention criterion used in stepwise regression analysis.** In (A-D) the blue color represents the OvCa dataset, green color represents the LuMe dataset, magenta color represents the LLML dataset and orange color represents the BrCa dataset.

**A. The Number of biomarkers retained**

**B. The Snedecor’s F statistic**

**C. Adjusted R^2^**

**D. Adjusted R^2^/Biomarker retained**

Figure A3**.** Distribution of model-fit R^2^ values in the 72 samples in which at least one biomarker was falsely associated with the disease status using randomly generated 1000 datasets each comprising 50 cases, 50 controls and 100 biomarkers.

Figure A4**. Influence of training set selection on the estimates of area under the ROC curve**

1. Multiple training set sampled from the OvCa dataset (number of samples = 100). The panel shows the results of factor analysis using the principal factors. The magenta colored dots and line (third order polynomial regression fit) indicates that with increasing size of the randomly sampled training set increasing number of training set load onto a common factor. Conversely, the blue colored dots and line indicates that the uniqueness of the samples decreases almost exponentially with increasing size of the sampled training set. This indicates that larger samples (exceeding a size of 100 for the OvCa dataset) even if chosen randomly are likely to yield similar pattern of area under the ROC curve for all the biomarkers.

1. For each training set sampled, we estimated the Spearman correlation coefficient between the area under the ROC curve in the random sample and the area under the ROC curve in the training set used for analysis in the main text. As expected, we observed that increasing size of the training set was associated with a higher correlation with the training set in terms of the area under the ROC curve for the 15,154 biomarkers.

1. Bootstrap estimates of the area under ROC curve for each biomarker shown Section 2.

**OvCa data set**

***Biomarker AUC BS-AUC 95% CI for BS-AUC***

v2668 0.8768 0.8840 0.8422 – 0.9393

v2310 0.0477 0.0486 0.0256 – 0.0881

v183 0.1290 0.1522 0.0846 – 0.2331

v2236 0.9877 0.9877 0.9722 – 0.9978

v700 0.1362 0.1379 0.0889 – 0.1921

v1680 0.0140 0.0143 0.0011 – 0.0478

v9607 0.1315 0.1315 0.0797 – 0.2090

v9606 0.1338 0.1338 0.0812 – 0.2670

v2309 0.0751 0.0751 0.0333 – 0.1274

v1674 0.1035 0.1071 0.0432 – 0.1605

v1594 0.0841 0.0862 0.0436 – 0.1589

v545 0.0841 0.0841 0.0342 – 0.1356

v576 0.1288 0.1295 0.0857 – 0.2041

v543 0.0728 0.0721 0.0323 – 0.1302

v546 0.1043 0.1043 0.0592 – 0.1699

v2308 0.1362 0.1397 0.0906 – 0.2594

v9609 0.1333 0.1333 0.0778 – 0.1954

v2311 0.0433 0.0436 0.0137 – 0.0790

**LuMe data set**

***Biomarker AUC BS-AUC 95% CI for BS-AUC***

v1136 1.0000 1.0000 1.0000 – 1.0000

v11368 1.0000 1.0000 1.0000 – 1.0000

v9863 0.0000 0.0000 0.0000 – 0.0000

v2549 0.0000 0.0000 0.0000 – 0.0000

v4336 0.0078 0.0083 0.0000 – 0.0504

**LLML data set**

***Biomarker AUC BS-AUC 95% CI for BS-AUC***

v1745 0.0236 0.0236 0.0000 – 0.0690

v6539 0.0606 0.0606 0.0000 – 0.1313

v1834 0.0202 0.0218 0.0000 – 0.0988

**BrCa data set**

***Biomarker AUC BS-AUC 95% CI for BS-AUC***

v13366 0.0799 0.0799 0.0415 – 0.1183

v7300 0.1928 0.1921 0.1116 – 0.2740

v5261 0.1379 0.1364 0.0783 – 0.1945

v15932 0.1230 0.1236 0.0715 – 0.1757

v22068 0.1942 0.1942 0.1315 – 0.2569

AUC – Area under ROC curve in the training set used for analysis in the main text, BS-AUC – Bootstrap estimate of the area under the ROC curve obtained after 500 replicates, CI – confidence interval corrected for bootstrap sampling bias.
